# High Osmolality Vitrification: A New Method for the Simple and Temperature-Permissive Cryopreservation of Mouse Embryos

**DOI:** 10.1371/journal.pone.0049316

**Published:** 2013-01-16

**Authors:** Keiji Mochida, Ayumi Hasegawa, Ming-Wen Li, Martin D. Fray, Seiji Kito, Jadine M. Vallelunga, K. C. Kent Lloyd, Atsushi Yoshiki, Yuichi Obata, Atsuo Ogura

**Affiliations:** 1 RIKEN BioResource Center, Tsukuba, Ibaraki, Japan; 2 Mouse Biology Program, School of Veterinary Medicine, University of California Davis, Davis, California, United States of America; 3 Medical Research Council Mary Lyon Centre, Harwell Science and Innovation Centre, Oxon, United Kingdom; 4 National Institute of Radiological Sciences, Chiba-shi, Chiba, Japan; 5 Graduate School of Life and Environmental Science, University of Tsukuba, Ibaraki, Japan; 6 The Center for Disease Biology and Integrative Medicine, Faculty of Medicine, University of Tokyo, Tokyo, Japan; University of Connecticut, United States of America

## Abstract

Procedures for cryopreserving embryos vary considerably, each having its specific advantages and disadvantages in terms of technical feasibility, embryo survival yield, temperature permissibility and species- or strain-dependent applicability. Here we report a high osmolality vitrification (HOV) method that is advantageous in these respects. Cryopreservation by vitrification is generally very simple, but, unlike slow freezing, embryos should be kept at a supercooling temperature (below –130°C) to avoid cryodamage. We overcame this problem by using an HOV solution containing 42.5% (v/v) ethylene glycol, 17.3% (w/v) Ficoll and 1.0 M sucrose. This solution is more viscous than other cryopreservation solutions, but easy handling of embryos was assured by employing a less viscous equilibration solution before vitrification. Most (>80%) embryos cryopreserved in this solution survived at –80°C for at least 30 days. Normal mice were recovered even after intercontinental transportation in a conventional dry-ice package for 2–3 days, indicating that special containers such as dry shippers with liquid nitrogen vapor are unnecessary. The HOV solution could also be employed for long-term storage in liquid nitrogen, as with other conventional cryoprotectants. Finally, we confirmed that this new vitrification method could be applied successfully to embryos of all six strains of mice we have tested so far. Thus, our HOV method provides an efficient and reliable strategy for the routine cryopreservation of mouse embryos in animal facilities and biomedical laboratories, and for easy and cheap transportation.

## Introduction

With the advent of molecular genetics and modern developmental biology, thousands of new mutant strains of mice have been generated by genetic modification or induced mutagenesis. However, it has become impossible to keep all mouse strains as breeding colonies in many facilities and now many of them are kept frozen as embryos in liquid nitrogen (LN_2_) for later use. Cryopreservation of embryos is a strategy not only for saving costs and space, but also for avoiding the microbiological and genetic contamination of valuable mouse strains. Since the first report of successful mouse embryo cryopreservation by slow freezing in 1972 [1], many technical improvements have been made to increase the survivability of embryos and to reduce the labor required during the freezing/thawing procedures [2]. At present, there are several embryo cryopreservation techniques, but each has specific disadvantages in terms of technical demands, embryo survival yield, temperature permissibility and applicability. Currently, vitrification is the technique of first choice for mouse embryo cryopreservation because of its technical simplicity, but it is still under intense research pressure for improvements. In general, vitrification requires neither slow cooling nor a programmable freezer, so the procedure is very rapid [3]. However, to avoid cryodamage to embryos, they should be kept supercooled below –130°C during cryopreservation and need to be warmed rapidly at recovery [3], [4]. If embryos are allowed to stay above the devitrification temperature, intracellular ice crystal formation occurs and this damages the embryos irreversibly. Therefore, when vitrified embryos are transported, they should be placed in a dry shipper: a large Dewar flask designed for the shipment of specimens in LN_2_ vapor. Dry shippers are heavy, bulky and expensive and the cost of a single transportation including an empty shipper return trip is high. This has been one of the biggest drawbacks of embryo cryopreservation by vitrification in laboratories and mouse strain depositories.

We have developed a new vitrification solution that has a higher osmolality than the existing vitrification solutions used to prevent devitrification of the cytoplasm at around –80°C [3]. The osmolality of the solution is about 19–28 mol/kg water, provided by high concentrations of ethylene glycol (EG) and sucrose. With this method, embryos are less susceptible to damage during handling and can be stored at –80°C for at least 10 days. Thus, while this new vitrification method has clear advantages over others, it was originally optimized for plastic straws, which are more fragile and less often used than the cryotubes for conventional cell freezing. In addition, the vitrification solution is highly viscous and requires some skill and experience in handling, as the embryos tend to float in it. This might hamper its conventional use in laboratories. Therefore, we sought to refine this vitrification method for easier use by mouse facility staff not specializing in embryo manipulation. We also examined the feasibility of our new protocol for international transportation of embryos using a dry-ice package instead of a dry shipper. Finally, we assessed its applicability for future broader use in mouse genetics by applying it to embryos from six different strains of mice.

## Results

### Optimization of the Composition of the Vitrification Solution Using Cryotubes

Embryos from the C57BL/6JJcl strain were used throughout the experiments unless otherwise stated. In the first series of experiments, we optimized the concentrations of the chemicals in the vitrification solution by assessing the survivability of embryos after vitrification in cryotubes at –80°C. First, we performed experiments with a fixed concentration of 40% (v/v) EG and 18% (w/v) Ficoll and altered concentrations of sucrose from 0.3 to 1.35 M (Table 1). Embryos were stored in either vitrification solution in cryotubes at about –80°C (with dry ice) for 2 days and then stored in LN_2_ until warming. After being warmed and retrieved in culture medium, no embryos were alive when they had been vitrified with the solutions containing 0.3 or 0.6 M sucrose (Table 2). Because the 0.3 M sucrose solution containing ethylene glycol and Ficoll (EFS; Ethylene glycol–Ficoll–Sucrose) is the vitrification solution we routinely used for mouse embryo cryopreservation (EFS40; for composition, see Table 1), this result reinforces the notion that the typical vitrification solutions used for storage in LN_2_ are inappropriate for storage at –80°C. The highest survival rate was obtained with 1.05 M sucrose, and 90% of the embryos developed into morulae or blastocysts subsequently (Table 2). These rates were not significantly different from those in the control group (*P*>0.05; Table 2).

**Table 1:** The composition and osmolality of the EFS types used in this study.

Solution*	Ethylene glycol (% v/v)	Sucrose (M)	Ficoll (% w/v)	Osmolality(mol/kg water)
EFS20	20	0.4	24	7.4
EFS35c	35	0.975	19.5	23.3
EFS40	40	0.3	18	18.0
EFS40c	40	0.9	18	28.0
EFS42.5c	42.5	1.01	17.3	35.1
EFS45c	45	0.825	16.5	33.6
EFS50c	50	0.75	15	40.3

* The number after ‘EFS’ denotes the concentration of ethylene glycol and ‘c’ means higher concentrations of sucrose (see ref. 3).

**Table 2:** Survivability and developmental ability of embryos vitrified and stored in 40% (v/v) ethylene glycol solution containing different concentrations of sucrose at –80°C for 2 days.

	No. of embryos				
Concentration of sucrose (M)	Vitrified	Retrieved (%)	Alive (%)	≥ 4 cell stage (%) among cultured embryos	Morulae or blastocysts (%) among cultured embryos
0.3	60	59 (98)	0 (0)^a^	–	–
0.6	60	60 (100)	0 (0)^a^	–	–
0.75	60	59 (98)	19 (32)^b^	18 (95)	14 (74)
0.9	60	60 (100)	41 (68)^b^	41 (100)	26 (63)^a^
1.05	60	59 (98)	50 (83)^bc^	48 (96)	45 (90)^b^
1.2	60	60 (100)	47 (78)^b^	47 (100)	33 (70)^a^
1.35	60	59 (98)	41 (68)^b^	40 (98)	22 (54)^a^
0.3 (Control)	60	60 (100)	57 (95)^c^	56 (98)	56 (98)^b^

The vitrification solutions also contained 18% (w/v) Ficoll.

Embryos in the control group were vitrified and stored in LN2.

Numbers with different superscripts within the same column are significantly different (P<0.05; by Chi-squared test with Yates’ correction).

Next, we examined the effect of the EG concentration by adding different volumes of EG (35% to 50%) to a solution containing 0.9 M sucrose (calculated based on 40% EG and 18% Ficoll). In this experiment, the concentration of sucrose and the solution’s osmolality were both changed according to the amount of EG added to the solution (Table 3). Embryos were stored in each vitrification solution in cryotubes at about –80°C (with dry ice) for 2 days and then stored in LN_2_ until warming. Embryos that were stored directly in LN_2_ without storage at –80°C were used as controls. The results are summarized in Table 3. When embryos were vitrified and stored at –80°C for 2 days, none in the 35% EG group were alive after warming. The highest survival rate (97%) was achieved with 45% EG, and this was not significantly different from the control (100%). Modest survival rates (68–80%) were obtained with 40% and 50% EG, but they were significantly lower than in the controls (*P*<0.05).

**Table 3:** Survivability and developmental ability of embryos vitrified and stored in 0.9 M sucrose and 18% (w/v) Ficoll solution* containing different concentrations of sucrose at –80°C for 2 days.

				No. of embryos			
Percentage of ethylene glycol (v/v)	Conc. of sucrose (M)	Final osmolality (mol/kg water)	Storage at –80°C for 2 days	Vitrified	Retrieved (%)	Alive (%)	≥ 4 cells (%) among cultured embryos
35	0.975	23.3	–	60	58 (97)	58 (100)^a^	58 (100)^a^
			+	60	60 (100)	0 (0)^b^	0 (0)
40	0.9	28	–	60	59 (98)	59 (100)^a^	58 (98)^a^
			+	60	60 (100)	41 (68)^c^	41 (100)^a^
45	0.825	33.6	–	60	59 (98)	59 (100)^a^	59 (100)^a^
			+	60	59 (98)	58 (97)^a^	57 (98)^a^
50	0.75	40.3	–	60	57 (95)	54 (90)^a^	18 (33)^b^
			+	60	60 (100)	48 (80)^b^	35 (73)^c^

* Concentrations of sucrose and Ficoll are based on a solution containing 40% ethylene glycol.

Numbers with different superscripts within the same column are significantly different (P<0.05; by Chi-squared test with Yates’ correction).

We undertook further fine optimization by extending the storage time to 160 days because the previous method by Jin et al. [3] cannot maintain the viability of embryos for more than 30 days at –80°C (Edashige and Kasai, unpublished). The concentration of EG was adjusted to 42.5%, 45%, or 50%. About 80% of embryos survived after a 30-day storage at –80°C in the 42.5% and 45% EG groups. After culture *in vitro*, about 90% or more developed to the morula or blastocyst stage in the 42.5% and 45% EG groups (Table 4). In the 45% EG group, about half of the embryos transferred developed to term offspring after a 2- or 7-day storage. Even after storage for 60 and 160 days with 45% EG, the survival rates remained good at 58% and 53%, respectively (Table 4). At term, 32% and 12%, respectively, of the transferred embryos from these experiments developed to normal offspring, although the latter outcome was significantly lower than for embryos stored for 2 or 7 days (*P*<0.05; Table 4), Nevertheless, the results indicated that live mice can be recovered from vitrified embryos stored at –80°C for about half a year. By contrast, embryos that had been vitrified with 50% EG showed significantly lower survivability after warming (*P*<0.05). Based on the results obtained above, we used the 42.5% EG or 45% EG solutions with high sucrose levels (termed EFS42.5c and EFS45c, respectively; see Table 1) for subsequent experiments.

**Table 4:** Survivability and developmental ability of vitrified embryos stored in different EFS solutions at –80°C for 160 days.

				No. of embryos							
Percent of ethylene glycol (v/v)	Conc. of sucrose (M)	Final osmolality (mol/kg water)	Days stored at –80°C	Vitrified	Retrieved (%)		Alive (%)	≥ Morulae (%)	Transferred	Implanted (%)	Developed to offspring (%)
42.5	1.006	35.1	2	60	57 (95)		57 (100)^a^	51 (89)^ab^			
			7	60	60 (100)		60 (100)^a^	58 (97)^a^			
			30	60	60 (100)		46 (77)^b^	42 (91)^ac^			
45	0.825	33.6	2	60	59 (98)		58 (98)^a^	51 (91)^ac^	36*	33 (92)^a^	17 (47)^a^
			7	60	58 (97)		54 (93)^a^	51 (94)^ac^	48*	42 (88)^a^	24 (50)^a^
			30	60	58 (97)		48 (83)^b^	46 (96)^a^			
			60	60	59 (98)		34 (58)^c^	**	28	16 (57)^b^	9 (32)
			160	50	49 (98)		26 (53)^c^	**	26	11 (42)^b^	3 (12)^b^
50	0.75	40.3	2	60	60 (100)		48 (80)^b^	35 (73)^bc^			
			7	60	59 (98)	13 (22)^d^		9 (69)^c^			

* The embryos transferred were different from those used for in vitro culture experiments.

** Surviving 2-cell embryos were transferred into the oviducts of pseudopregnant recipient females.

Numbers with different superscripts within the same column are significantly different (P<0.05 by Chi-squared test with Yates’ correction).

### Transportation Experiments Using EFS45c

In the next series of experiments, we examined the feasibility of transporting embryos vitrified in EFS45c in a dry-ice package. In the first trial, we tested the survivability and developmental ability of embryos after domestic transportation. As a control experiment, we stored vitrified embryos at –80°C for 20 h and then immersed them in LN_2_ until the time of embryo transfer. All embryos in the control group were alive after warming and 78% of the transferred embryos developed to live pups (Table 5). In the domestic transportation experiment, embryos were transported in a dry-ice package and then transferred into LN_2_ in the recipient facility until recovered. The embryos were kept in the dry-ice package for 48 h including transportation. After warming, 99% of the embryos were alive and 78% reached term after embryo transfer, indicating that transportation of embryos in a dry-ice package did not affect their viability adversely (Table 5). The health status of these pups is summarized in Table S1.

**Table 5:** Survivability and developmental ability of embryos after domestic transportation with dry ice.

	No. of embryos				No. of embryos		
Transportation	Vitrified	Retrieved (%)	Alive (%)	No. of recipients pregnant/used (%)	Transferred	Implanted (%)	Developed to offspring (%)
–	100	99 (99)	99 (100)	5/5 (100)	59	54 (92)	46 (78)
+	125	124 (99)	123 (99)	9/10 (90)	123*	103 (93)*	74 (67)*

* Among pregnant recipients.

The time of storage in a dry-ice package was 20 h and 48 h for holding and transportation experiments, respectively.

For international transportation experiments, we shipped vitrified embryos from RIKEN BioResource Center (Japan) to the United States or the United Kingdom with dry ice. It took 2 days and 3 days before the packages arrived at the recipient facilities, respectively. Soon after delivery, the embryos were transferred into LN_2_. After warming, more than 90% of the embryos looked normal and more than 40% reached term after embryo transfer (Table 6). The pups were all normal in appearance. All the offspring examined as adults were fertile as far as we examined (n = 8).

**Table 6:** Embryo survival rates and developmental ability after international transportation with dry ice.

	No. of embryos				No. of embryos		
Transportation	Sent	Retrieved (%)	Alive (%)	No. of recipients pregnant/used (%)	Transferred	Implanted (%)	Developed to offspring (%)
From Japan to the USA	100	100 (100)	99 (99)	5/5 (100)	97	70 (72)	47 (48)
From Japan to the UK	75	67 (89)	61 (91)	2/2 (100)	43	–*	17 (40)

* Not observed.

### Modification of the Protocol for Improved Embryo Handling Before and After Vitrification

**Figure 1:**
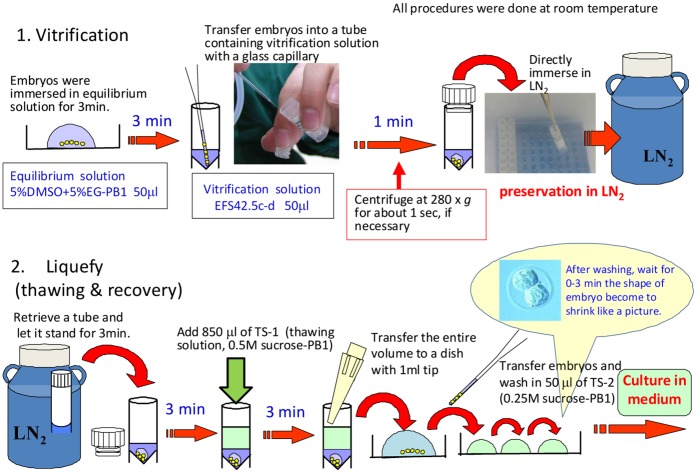
The overall scheme of the HOV method including vitrification and warming of embryos.

As described above, we developed a vitrification solution that allowed us to cryopreserve embryos at –80°C for several months and to transfer them in a dry-ice package. However, because the vitrification solution developed was very viscous and had a very high osmolality, embryos were often lost before or after vitrification unless the operators had developed enough experience in using this solution. To improve the ease of handling of embryos during the vitrification procedures, we further refined the protocol as follows. First, we added dimethyl sulfoxide (DMSO) to the equilibration solution to decrease the viscosity so that embryos could settle on the bottom of the dish. This enabled quick and easy recovery of embryos from the equilibration solution so that the equilibration time could be controlled precisely. We tested two equilibration solutions as follows: 5% DMSO and 5% EG in PB1 (5D5E-PB1) and 8% DMSO and 8% EG in PB1 (8D8E-PB1). First, fresh embryos were exposed to either of the equilibration solutions for 1, 3 or 5 min and then their survivability and ability to develop to the blastocyst stage were examined. As shown in Table S2, exposure to 5D5E-PB1 did not significantly affect the developmental potential of embryos within the time range tested. Therefore, this solution was used as an equilibration solution in the subsequent experiments. Next, we tested whether embryos vitrified by our new method could be allowed to warm slowly at room temperature so that we could avoid the addition of a warming solution to the tube, as is used for rapid warming in the current vitrification protocol using EFS40. This is because, without practice, and depending on the operator, addition of the warming solution sometimes causes fluctuations in the warming speed. In this experiment, embryos in all groups but one were stored at –196°C because we also examined the effects of slow warming on the survival of embryos cryopreserved using our conventional vitrification solution. Although embryos vitrified by our routine protocol using EFS20 and EFS40 solutions survived the standard rapid warming at a high rate (98%), only 8% survived slow warming at room temperature (Table 7). By contrast, 98% and 96% of embryos vitrified by the new method were alive after rapid and slow warming, respectively. The surviving embryos could develop into morulae and blastocysts at practical rates irrespective of the warming protocol. Embryos vitrified by our new protocol and stored at –80°C showed similar results (Table 7). Embryo transfer experiments revealed that about 60% of the embryos developed into offspring in all experimental groups, indicating that exposure to equilibration–vitrification or vitrification–warming media did not affect the development of embryos *in vivo* (Table S3). We also confirmed that these findings were also the same for another major mouse strain, BALB/cA (Table 7). The overall scheme of the finally optimized protocol is shown in Fig. 1. As shown in this figure, we recommend a short-time centrifugation (280× *g*) of cryotubes immediately before storage in LN_2_ if the equilibration medium is kept floating on the surface of the vitrification medium. This assures more consistent embryo survival after thawing.

**Table 7:** Survivability and developmental ability of embryos vitrified and warmed by different methods.

				No. of embryos				
Strain of embryos	Equilibrium solution	Vitrification solution	Warming method	Vitrified	Retrieved (%)	Alive (%)	≥ 4-cell stage (%)	Morulae or blastocysts (%)
C57BL/6J	EFS20	EFS40	Rapid*	120	120 (100%)	117 (98%)	114 (97%)	112 (96%)
			Slow	120	120 (100%)	10 (8%)***	10 (100%)	10 (100%)
	5D5E-PB1	EFS42.5c	Rapid	110	109 (99%)	107 (98%)	106 (99%)	104 (97%)
			Slow	110	110 (100%)	106 (96%)	102 (96%)	99 (93%)
			Slow**	60	57 (95%)	53 (93%)	47 (89%)	46 (87%)
BALB/cA	EFS20	EFS40	Rapid	120	118 (98%)	97 (82%)	90 (93%)	77 (79%)
			Slow	120	120 (100%)	20 (17%)***	17 (85%)	12 (60%)
	5D5E-PB1	EFS42.5c	Rapid	120	118 (98%)	110 (93%)	104 (95%)	83 (75%)
			Slow	120	120 (100%)	116 (97%)	112 (97%)	81 (70%)

* Current major protocol by vitrification with EFS20–EFS40 followed by rapid warming.

** Storage at –80°C for 2 days. Embryos in other groups had been stored in LN2 before warming.

*** Significantly different (P<0.05 by Chi-squared test with Yates’ correction) from the corresponding rapid warming group. For compositions of the EFS types used, see Table 1.

Lastly, we examined whether our vitrification protocol optimized using C57BL/6JJcl embryos could be applied to embryos from other strains of mice. As shown in Table 8, at least 93% of embryos showed normal morphology after recovery and 30–82% developed to term offspring after embryo transfer, irrespective of the strain used.

**Table 8:** Survivability and developmental ability of vitrified embryos in six strains of mice.

	No. (%) of embryos				No. of embryos		
Strain	Vitrified	Retrieved (%)	Alive (%)	No. of recipients pregnant/used (%)	Transferred	Implanted (%)	Developed to offspring (%)
C57BL/6J	265	263 (99)	256 (97)	3/3 (100)	39	36 (92)	32 (82)
C57BL/6N	175	173 (99)	168 (97)	3/3 (100)	40	36 (90)	21 (53)
BALB/cA	210	210 (100)	206 (98)	3/3 (100)	40	31 (78)	18 (45)
129/SvJ	100	100 (100)	93 (93)	3/3 (100)	41	33 (80)	27 (66)
DBA/2N	200	200 (100)	193 (97)	6/6 (100)	77	44 (57)	25 (32)
C3H/HeN	100	99 (99)	96 (97)	3/3 (100)	41	27 (66)	19 (46)

## Discussion

In this study, we have developed a new vitrification method for mouse embryos that enabled us to store the embryos at –80°C for several months and to transport them safely in dry-ice packages even over intercontinental distances. The vitrification solution we developed was a modification of an ethylene glycol-based solution that was originally developed by Kasai et al. in 1990 [5]. Ethylene glycol is less toxic than other cryoprotectants such as DMSO and propylene glycol and has been used widely for the vitrification of embryos and oocytes from various mammalian species including the rat [6], rabbit [7], cow [8], horse [9], sheep [10], Mongolian gerbil [11] and the multimammate mouse, *Mastomys* [12]. Unlike slow freezing methods, the success of vitrification methods depends critically on the rate of temperature change during cooling and warming. Therefore, for most vitrification methods developed so far, plastic straws have been preferred as containers because the temperature change outside a plastic straw is transmitted rapidly to the medium inside through its thin wall. This was also the case with the most recent vitrification method reported by Jin et al. [3]. However, plastic straws are fragile and need a certain skill level in handling during freezing and thawing. Therefore, they are less often used in laboratories than the cryotubes conventionally used for cell freezing. Here, we have optimized the vitrification method developed by Jin et al. [3] for efficient embryo cryopreservation using cryotubes so that the embryos could be cryopreserved more conveniently and transported for long distances more safely. Similarly, we have developed a freezing method for mouse spermatozoa optimized for cryotubes instead of plastic straws [13]. Nakao et al. [14] previously developed an embryo vitrification method using cryotubes, but it needed a specific combination of a cooling device with a cryotube to keep the temperature inside the tubes at 0°C during storage. The method presented in this study allows handling of embryos at ambient temperature throughout procedures.

As a consequence of our modification of the vitrification solution, its osmolality was increased from 18.0 mol/kg water to 33.6 or 35.1 mol/kg water and became more viscous. This caused the embryos to float near the surface of the solution during equilibration and made their handling difficult. This was a potential shortcoming of our modified vitrification method and is common to other EG-based vitrification methods. We found that this could be overcome by using an equilibration solution containing DMSO to reduce the viscosity. The mixture of DMSO and EG was first used successfully as a medium for the equilibration and vitrification of mouse blastocysts [15]. More recently, it was also used as an equilibration medium for vitrification using an open-pulled straw method based on DMSO, EG, Ficoll and sucrose [16]. The present study suggested that the same mixture worked well as an equilibration medium for DMSO-free vitrification. We also found that the embryos vitrified by our method could be warmed more slowly than those vitrified by the existing method, probably thanks to the temperature-permissiveness of our protocol. Thus, our new vitrification protocol overcame the shortcomings inherent in conventional vitrification methods–namely the requirement for precise control of the temperature during storage and of the timing during cooling/warming–while providing technical ease and simplicity of use. We termed our new vitrification method high osmolality vitrification (HOV). HOV was devised specifically for 2-cell mouse embryos and is therefore not applicable to mouse oocytes, which are known to be very sensitive to vitrification [4]. To circumvent this problem, minimal volume vitrification approaches with open containers such as the Cryotop and Croleaf methods have been developed. As far as we examined, these open containers are not suitable for storage at –80°C because the temperature of the solution increases quickly to above that point during handling, resulting in irreversible damage to the embryos.

Besides such technical advantages, HOV has important implications for the operation of mouse strain depository facilities in its security and cost-effectiveness. Our center and other major mouse depository centers in the world constitute the Federation of International Mouse Resources (FIMRe), a worldwide group that is responsible collectively for the safe maintenance of a number of invaluable mouse strains. Once they are cryopreserved by vitrification, the embryos should be kept below –130°C, usually relying on a continuous supply of LN_2_ so that devitrification can be avoided. We expect that embryos vitrified by HOV might survive the accidental loss of LN_2_ or breakage of tanks if they could be evacuated safely into deep freezers or dry-ice packages. This is an important advantage over the method reported by Jin et al. [3], which cannot be applied to long-term storage (e.g., 30 days) at –80°C (Edashige and Kasai, personal communication). The high applicability of HOV to different strains of mice would also increase the possibility of rescuing embryos (Table 8). Importantly, HOV-frozen embryos do not need bulky dry shippers for transportation and this could accelerate the exchange of mouse strains between mouse facilities. We estimate that the shipping costs of embryos in dry-ice packages might be reduced by from one-half to one-fourth of the costs incurred by dry shippers. In this study, we confirmed that vitrified embryos could be transported not only domestically but also intercontinentally: from our center in Japan to the University of California, Davis, in the USA or to the MRC Mammalian Genetics Unit in Harwell in the UK; both of which are members of FIMRe. Thus, the HOV technique is a promising candidate to become the standard method for mouse embryo cryopreservation.

In conclusion, we have devised a novel vitrification method for mouse embryos. It has several important advantages as follows. 1) The cooling process is simple and quick. 2) The warming process can be performed slowly, as in the slow freezing method. 3) Vitrification can be done at room temperature and does not need a programmable cell freezer. 4) Mouse embryos can survive even at –80°C for several months and can be transported internationally on dry ice. 5) The 2-cell embryos of six major inbred strains can be preserved stably with high survival rates (93–100%) with a good ability to develop into offspring (33–82%). Thus, HOV is eminently applicable for routine embryo cryopreservation in many mouse facilities and biomedical laboratories.

## Materials and Methods

### Animals

Mouse strains C57BL/6JJcl, C57BL/6NJcl, BALB/cAJcl, C3H/HeNJcl (CLEA Japan Inc., Tokyo, Japan), 129/SvJJmsSlc (SLC Co. Ltd., Shizuoka, Japan), and DBA/2NCrlCrlj (Charles River Lab. Japan Inc.) were used to produce embryos by *in vitro* fertilization. For embryo transfer experiments, ICR-strain female mice (CLEA Japan Inc.) were used as pseudopregnant recipients after being mated with vasectomized ICR-strain male mice. All mice were maintained under specific-pathogen-free conditions, provided with water and commercial laboratory mouse chow *ad libitum* and housed under controlled lighting conditions (light: 0700–2100). All animal experiments described here were approved by the Animal Experimentation Committee at the RIKEN Tsukuba Institute and were performed in accordance with the committee’s guiding principles.

### *In Vitro* Fertilization

This was performed as described [17]. In brief, spermatozoa from the epididymal caudae of male mice (12–16 weeks old) were suspended in 450 µl of human tubal fluid (HTF) medium [18] containing 0.3% bovine serum albumin (Merck Biosciences, San Diego, CA, USA) and hypotaurine (0.11 mg/ml, Sigma-Aldrich, St Louis, MO, USA) covered with sterile silicone oil (Sigma-Aldrich). They were preincubated at 37°C under 5% CO_2_ in air for 1–2 h. Superovulation was induced in females of the same strain as the male by an injection of 7.5 IU pregnant mare serum gonadotropin (PMSG; Peamex, Sankyo Co., Tokyo, Japan), followed 48–50 h later by 7.5 IU human chorionic gonadotropin (hCG; Puberogen, Sankyo Co., Tokyo, Japan). Cumulus–oocyte complexes (20–30 per drop) were collected from the oviducts and placed into 80 µl drops of HTF medium under silicone oil.

Insemination was carried out by adding a small drop of sperm suspension to HTF drops (final sperm concentration of 2–3×10^5^ cells/ml). At 4–6 h after insemination, the eggs were removed from the fertilization drops, washed in glucose-containing CZB medium [19] and cultured overnight in 10 µl drops of CZB. Embryos that reached the 2-cell stage on the next day were used for cryopreservation experiments.

### Cryopreservation of Embryos

We used vitrification solutions containing EG (Wako Pure Chemical Industries, Ltd., Osaka, Japan), Ficoll (Ficoll PM-70, GE Healthcare, Bio-Sciences AB, Uppsala, Sweden) and sucrose in PB1 [20]; for the concentrations, see Table 1. In the initial series of experiments, we assessed the survivability of embryos cryopreserved in solutions with different concentrations of cryoprotectant or sucrose in cryotubes at –80°C, essentially based on a method reported previously [21]. Before cooling, 2-cell embryos were suspended in equilibrium solution (EFS20) for 2 min. Ten to 25 embryos were then picked up using a sterile glass capillary tube with a small amount of solution and transferred into cryotubes (MS-4501; Sumitomo Bakelite Co. Ltd., Tokyo, Japan), each containing 50 µl of the vitrification solution to be tested. After exposure to these solutions at room temperature for 1 min, the cryotubes were immersed directly into LN_2_. After the cryotubes had been exposed to –80°C for certain times or had been transported in a dry-ice package (described below), they were again stored in LN_2_ until warming. To thaw (liquefy) the vitrified embryos, the cryotubes were retrieved from LN_2_ and the cap was quickly removed to allow LN_2_ to evaporate from the tubes. After they had been left at room temperature for 30 sec, 850 µl of 0.75 M sucrose-PB1 was added to the tubes and the solution was mixed by gentle pipetting until it was dissolved evenly. Then the entire volume of the cryotube was transferred to a plastic dish. After equilibration for 3 min, the embryos were picked up using a glass capillary tube and transferred into a 0.25 M sucrose-PB1 drop. After equilibration for another 3 min, the embryos were transferred into a drop of 0.25 M sucrose-PB1 and then to a drop of CZB medium in a culture dish. Embryos with normal morphology (i.e., with an intact plasma membrane and clear cytoplasm) were considered viable. The surviving embryos were incubated under 5% CO_2_ in air at 37°C. In a subsequent series of experiments, we tested equilibration solutions containing DMSO (MP Biomedicals LLC; Irvine, CA, USA; Cat # 194819): for the method, see Results. For slow warming, cryotubes were left standing at room temperature for 3 min and then 850 µl of 0.5 M sucrose-PB1 solution was added gently. Three minutes later, the solution was mixed gently by pipetting about three or four times and the entire volume was transferred to a plastic dish. After being washed in three new drops of 0.25 M sucrose-PB1, the surviving embryos were transferred to a drop of CZB medium and incubated under 5% CO_2_ in air at 37°C. This final protocol is shown in Figure 1. The protocol was also used for assessment of its applicability to embryos from different strains of mice (Table 8).

### Storage and Transportation of Vitrified Embryos at –80°C

Cryotubes containing vitrified embryos were retrieved from LN_2_ and transferred to a zippered aluminum bag (Rami zip AL-10, Seisan Nippon Sha Ltd., Tokyo, Japan) above the surface of the LN_2_ so that the temperature could be kept below –80°C. Before closing the bag, excess air was squeezed out as much as possible. For storage for 1 week or less, the aluminum bag was placed near the center of a styrene foam box filled with dry-ice pellets (about 10 mm diameter ×30 mm long) so that the bag was completely covered with dry ice. The box was placed in a freezer at –40°C. For storage over more than 1 week, the aluminum bag was placed in a freezer at –80°C. The box containing the embryos was transported using a commercial courier service.

### Statistical Analysis

The data were analyzed statistically using Chi-squared tests with Yates’ correction and *P*<0.05 was considered statistically significant.

## Supporting Information

Table S1 Status of the offspring born after domestic transportation experiments.
(DOC)

Table S2 Developmental ability of embryos equilibrated in different solutions at room temperature.
(DOC)

Table S3 Development of embryos *in vitro* and *in vivo* after exposure to the medium or to vitrification.
(DOC)
