# Supplementary material for: High Osmolality Vitrification: A New Method for the Simple and Temperature-Permissive Cryopreservation of Mouse Embryos
Source: PLoS One. 2013 Jan 16;8(1):e49316. doi: 10.1371/journal.pone.0049316 (PMC3547031; doi:10.1371/journal.pone.0049316)
Supplement: Table S1 — Status of the offspring born after domestic transportation experiments. (DOC) [file pone.0049316.s001.doc]

| Supplementary Table S1. Status of the offspring born after domestic transportation experiments (see Table 4). | | | | | | | |
| --- | --- | --- | --- | --- | --- | --- | --- |
| Transportation | Gender | No. of offspring | | |  | Body weight | |
|  |  | Total | Alive with normal morphology | Abnormal |  | Mean ± S.E. (g) | Range (g) |
| – | Female | 21 | 20 | 1* |  | 1.45 ± 0.02 | 1.26–1.65 |
|  | Male | 25 | 25 | 0 |  | 1.47 ± 0.03 | 1.11–1.64 |
| + | Female | 27 | 26 | 1** |  | 1.34 ± 0.02 | 1.12–1.48 |
|  | Male | 47 | 47 | 0 |  | 1.40 ± 0.02 | 0.99–1.65 |
| *Stillborn. | | | | | | | |
| **Small eyes. | | | | | | | |
