# Supplementary material for: High Osmolality Vitrification: A New Method for the Simple and Temperature-Permissive Cryopreservation of Mouse Embryos
Source: PLoS One. 2013 Jan 16;8(1):e49316. doi: 10.1371/journal.pone.0049316 (PMC3547031; doi:10.1371/journal.pone.0049316)
Supplement: Table S2 — Developmental ability of embryos equilibrated in different solutions at room temperature. (DOC) [file pone.0049316.s002.doc]

| Supplementary Table S2. Developmental ability of embryos equilibrated in different solutions at room temperature. | | | | | | | |
| --- | --- | --- | --- | --- | --- | --- | --- |
| Equilibrium solution | Time of equilibration (min) | No. of embryos | |  | Embryo status | | |
|  |  | Tested | Normal (%) |  | Morulae (%) | Blastocysts (%) | Expanded blastocysts (%) |
| 5D5E-PB1 | 1 | 60 | 60 (100%) |  | 60 (100%) | 60 (100%) | 58 (97%) |
|  | 3 | 60 | 60 (100%) |  | 60 (100%) | 59 (98%) | 55 (92%) |
|  | 5 | 60 | 60 (100%) |  | 59 (98%) | 54 (90%) | 51 (85%) |
| 8D8E-PB1 | 1 | 60 | 60 (100%) |  | 59 (98%) | 59 (98%) | 56 (93%) |
|  | 3 | 60 | 60 (100%) |  | 59 (98%) | 51 (85%) | 49 (82%) |
|  | 5 | 60 | 60 (100%) |  | 55 (92%) | 45 (75%) | 37 (62%)* |
| Control | 0 | 60 | 60 (100%) |  | 60 (100%) | 57 (95%) | 55 (92%) |
| *Significantly different from the control (*P* < 0.05; by Chi-squared test with Yates’ correction). | | | | | | | |
