# Supplementary material for: High Osmolality Vitrification: A New Method for the Simple and Temperature-Permissive Cryopreservation of Mouse Embryos
Source: PLoS One. 2013 Jan 16;8(1):e49316. doi: 10.1371/journal.pone.0049316 (PMC3547031; doi:10.1371/journal.pone.0049316)
Supplement: Table S3 — Development of embryos in vitro and in vivo after exposure to the medium or to vitrification. (DOC) [file pone.0049316.s003.doc]

| **Supplementary Table S3. Development of embryos *in vitro* and *in vivo* after exposure to the medium or to vitrification.** | | | | | | | | | | |
| --- | --- | --- | --- | --- | --- | --- | --- | --- | --- | --- |
| Treatment | No. of embryos | | | | | | No. of recipients pregnant /used (%) | No. of embryos | | |
|  | Used | Retrieved (%) | Alive (%) | Cultured | Morulae (%) | Blastocysts (%) |  | Transferred** | Implanted (%) | Developed to offspring (%) |
| Control | 176 | - | - | 110 | 110 (100) | 103 (94) | 5/5 (100) | 66 | 55 (83) | 40 (61) |
| Exposure* | 103 | 102 (99) | 100 (98) | 48 | 47 (97) | 45 (94) | 4/4 (100) | 52 | 48 (92) | 32 (62) |
| Vitrification | 113 | 109 (96) | 107 (98) | 55 | 55 (100) | 48 (87) | 4/4 (100) | 52 | 40 (77) | 31 (60) |
| *Embryos were exposed to 5D5E-PB1 followed by EFS42.5c. | | | | | |  |  |  |  |  |
| * *Two-cell embryos were transferred into oviducts of recipient females. | | | | | | |  |  |  |  |
